# Associations of park visits, physical activity and health outcomes among churchgoing Latino adults in low-income urban communities

**DOI:** 10.1007/s10865-026-00672-4

**Published:** 2026-05-06

**Authors:** Kathryn P. Derose, Rachana Seelam, Lilian G. Perez, Elva Arredondo, Bing Han, Gabriela Castro, Deborah A. Cohen

**Affiliations:** 1 RAND, Santa Monica, CA USA; 2 University of Massachusetts Amherst, Amherst, MA USA; 3 School of Public Health, San Diego State University, San Diego, CA USA; 4 Kaiser Permanente Research and Evaluation, Pasadena, CA USA

**Keywords:** Latino populations, Parks, Physical activity, Mental health, Faith-based organizations

## Abstract

This study examines factors associated with park visits, physical activity (PA), and chronic health conditions using baseline data from a cluster randomized controlled trial to promote PA and park visits among churchgoing Latino adults in low-income urban communities. Participants (*n* = 726) were recruited from 12 Catholic churches in predominantly Latino neighborhoods in and near East Los Angeles. Data collection included accelerometer-measured PA, self-reported weekly park visits, socio-demographics, history of hypertension and Type 2 diabetes, family and friend social support for PA, depression (PHQ8), stress, and measured height and weight. Multivariable linear and logistic regression models assessed associations between predictors and health outcomes. The average age was 52.3 years; 76% were women; 71% had completed high school or less; and the average BMI was 31.1 (obese). Age, female gender, and higher depressive symptoms were negatively associated with moderate-to-vigorous PA (MVPA). Being employed, greater depressive symptoms, and more safety concerns were negatively associated with park visits, while higher family support for PA was positively associated. Both accelerometer-based MVPA and self-reported park visits were associated with lower depressive symptoms. Park visits were also associated with lower odds of Type 2 diabetes, and higher family support for PA was linked with reduced odds of hypertension. These results address gaps in understanding the relationships between PA and park visits and physical and mental health among churchgoing Latino adults. Churches may serve as important venues to support family-oriented park events and promote greater use of parks for health promotion in Latino communities. Association of park visits, physical activity and health outcomes among Latino church members in low-income urban communities.

*Clinical trial registration number* NCT03858868; 2019-02-27

## Background

Urban public parks are vital community resources that support physical activity (PA), a key behavior promoting health and well-being (Dhuli et al., 2022). Studies consistently show that park use is associated with higher levels of PA and reduced prevalence of chronic conditions such as diabetes and heart disease (Fuente et al., 2020, Walker et al., 2022). Parks also foster positive mental health outcomes,^1,4^ as spending time in nature is linked to lower stress levels and reduced depressive symptoms (Deyo et al., 2024) as well as lower blood pressure, reduced nervous system arousal, enhanced immune system function, reduced anxiety and increased self-esteem (Sundermann et al., 2023). Despite these benefits, parks are often underutilized, particularly in high poverty, urban neighborhoods (Cohen et al., 2016, Cohen et al., 2012). The reasons for the underutilization are complex and include factors like higher crime (objective or perceived) and less programming (Cohen et al., 2012, Han et al., 2018) and vary by gender and structural characteristics of parks (Derose et al., 2019). This underuse highlights the need for strategies that will address these issues and encourage more visits and increase PA among these communities.

Latino^1^ populations comprise the largest racial ethnic minoritized population in the U.S. and are disproportionately represented in low-income communities. In addition, Latino adults are highly affected by chronic health conditions such as diabetes for which PA plays an important role in prevention and management (Colberg et al., 2016). Nevertheless, little is known about the specific PA and health benefits of park use among Latino populations in high-poverty areas. National studies have found that Latino populations report using parks with a similar frequency to other racial-ethnic groups but are more likely to visit the park with a child family member and/or someone else (i.e., not visit alone) (Vaughan et al., 2018). A large study of Los Angeles parks (*n* = 50) and community residents within a mile of each park (*n* = 7506) found this tendency for park-based social interactions among Spanish-speaking Latino populations but not English-speaking Latinos, suggesting that acculturation may play a role (Derose et al., 2015). Further, another Los Angeles study of parks (*n* = 48) and park users (*n* = 3213, 85% Latino) found significant gender differences with women using parks for PA less often than men, and this lower use was largely mediated through women’s higher perceptions of crime and a lack of walking paths in parks (Derose et al., 2019). Past research has also documented multiple barriers to park use among Latino populations, including limited time, safety concerns, as well as the lack of park programs, amenities and staff (Luna et al., 2024). Nevertheless, there is little research specifically examining the relationship between Latino populations’ park use and PA and health outcomes, which could inform interventions to improve Latino populations’ health behaviors (PA), mental health and chronic disease outcomes.

Faith-based organizations, including churches, have extensive reach, trust, and influence within Latino communities and thus can be effective partners in community health efforts, particularly Latino immigrant communities (Flórez et al., 2020; Derose and Rodriguez 2020). Even with overall declines in church attendance and affiliation in the US, a large portion of Latino populations still identify as Catholic (43%) or Protestant (21%) (Krogstad et al., 2025). Working with faith-based communities to conduct health-related research has become a popular strategy over the past few decades, including around PA (Tristão Parra et al., 2018), although relatively few faith-based health interventions have focused on Latino populations as compared to African American populations (Flórez et al., 2020; Derose and Rodriguez 2020). In addition, few studies with faith-based communities have examined park use among congregants (Arredondo et al., 2013; Perez et al., 2022; Derose et al., 2022; Perez et ai., 2024) or measured PA using accelerometry (Derose et al., 2022, Wilcox et al., 2010; Baruth et al., 2013, Arredondo et al., 2015). Latino populations have high levels of religious affiliation, thus exploring park use and PA among members of Latino faith communities and factors associated with these could inform future research that aims to increase park use and PA and improve health outcomes in this population.

This study fills existing gaps in the literature by examining factors associated with park visits, objectively-measured MVPA, and chronic health conditions among churchgoing Latino populations – a group with unique social and cultural dynamics influencing PA behaviors. We use baseline data from *Parishes & Parks*, a cluster randomized controlled trial to promote PA and park visits among churchgoing Latino adults (Derose et al., 2022). Specifically, we ask:

1. What are the factors associated with MVPA in this church-going Latino sample?
2. What are the factors associated with park use in this church-going Latino sample?
3. Are an individuals’ MVPA and park use associated with mental health (depression, perceived stress) and other health outcomes (obesity, diabetes, and hypertension) in this church-going Latino sample?

It is important to note that although park visits and PA often occur together, they may influence health through partly independent mechanisms. Park visits can reflect exposure to natural environments, opportunities for social interaction, and psychosocial restoration, each of which has been shown to reduce stress, improve mood, and lower cardiometabolic risk factors such as blood pressure and glucose regulation beyond total PA (White et al., 2019; Hartig et al., 2014; Twohig-Bennett & Jones 2018). In contrast, MVPA contributes primarily through physiological pathways linked to cardiovascular fitness and energy expenditure (Warburton and Bredin 2017, Piercy et al., 2018). Family support for PA has been found to be particularly important for leisure-time PA among middle-aged and older adults (Lindsay Smith et al., 2017) and may also affect health outcomes beyond its direct impact on PA. Supportive social relationships have been found to influence health through behavioral, psychosocial, and physiological mechanisms, which can positively influence stress regulation, blood pressure control and metabolic functioning independent of actual PA participation (Umberson and Karas Montez 2010). Including park visits, MVPA, and family support for PA in the models therefore allows for examination of their unique as well as overlapping associations with physical and mental health.

## Methods

### Participants

Participants were recruited as part of an RCT to promote PA from 12 Catholic churches from one Deanery in East Los Angeles and surrounding communities, which are predominantly (80–97%) Latino areas. In addition, these areas had high poverty levels, with the percent of families living in poverty within one mile of each church (using American Community Survey data) at the time of recruitment ranging from 17 to 38%. Full details on church and participant recruitment are provided in a published protocol paper (Derose et al., 2022). Parishioners from the participating churches were eligible to participate if they were 18 years of age or older, attended the church in the past 6 months and more than once a month, planned on attending the church for the next year and did not attend other participating churches, resided within 15 min driving distance of the church and planned to reside in the same neighborhood for the next year, and were able to participate in activities at the church during the week. We implemented the study across two cohorts among 12 churches. The first cohort completed baseline in 2019, before the beginning of the COVID-19 pandemic; given the extended delays that resulted from COVID-19 restrictions at churches and parks in Los Angeles, the second cohort’s baseline was not completed until 2023.

### Data collection

Baseline data collection was conducted at each of the 12 churches primarily on Sundays to make it easier for participants (at a time they were already at church). Data were collected using Open Data Kit questionnaires in English and Spanish on tablets. Trained bilingual research assistants (RAs) conducted biometric assessments and administered the health survey if participants requested (otherwise, health survey was self-administered). The preferred (and most common) procedure was to have participants complete survey and biometric measures upon enrollment and take the accelerometer to wear for 7 days and return the next Sunday. But to accommodate participants’ schedules, sometimes they took the accelerometer first and completed other measures when they brought it back. The research was reviewed and approved by the RAND IRB.

## Measures

*Accelerometry* (objectively measured PA). Participants were asked to wear an accelerometer for one week using ActiGraph GT3X attached to an elastic belt over their right hip for 7 consecutive days. Participants were instructed to wear the device for at least 12 h a day, every day and to remove it during water activities and sleep. Non-wear time was defined as 60 consecutive minutes of 0 count values (Migueles et al., 2017). For data processing using ActiLife v.6.13.4 software (ActiGraph), we defined valid wear time as 10 h or more a day and 3 or more valid days (Evenson et al., 2015). Files were downloaded as 60-second epochs and processed using the Freedson 1998 cut-points (Freedson et al., 1998), which define moderate-intensity as 1952–5724 counts per minute (CPM) and vigorous-intensity as 5725–9498 CPM. We calculated average minutes per day of MVPA using total days with valid data and then multiplied by 7 to estimate MVPA minutes per week.

*Season* (of accelerometry measure) was recorded to adjust for well-documented seasonal influences on PA, even in temperate climates (Tucker and Gilliland 2007; Turrisi et al., 2021; Garriga et al., 2022). Although Los Angeles has a temperate climate that permits year-round outdoor activity, modest fluctuations in temperature, daylight, and air quality can nevertheless influence activity patterns and device wear.

*Body Mass Index* (BMI). Trained RAs measured height and weight of participants. Participants were asked to stand on a Tanita digital scale wearing light clothing and without shoes to assess weight to the nearest 0.1 kg. Height was measured to the nearest 0.1 cm using a fixed ruler attached to Seca heigh board vertical surface; participants stood with their back flat against the vertical surface without shoes. We calculated BMI as kg/m^2^.

The remaining measures were collected via survey, including:

*Park use* and *perceived park safety* were measured using items from our previous studies (Derose et al., 2015; Evenson 2013), including number of park visits in the past 7 days and perceptions of park safety (*very safe*,* safe*,* not very safe*,* not safe at all*, with higher scores meaning less safe) (Cohen et al., 2016). 

*Mental health* was assessed using the 8-item version of the Patient Health Questionnaire (PHQ-8) (Kroenke et al., 2009) for depressive symptoms, which has been validated with individuals of Mexican American and Central American descent in the U.S. (Alpizar et al., 2018) and the 4-item Perceived Stress Scale 4 (PSS 4) (Cohen et al., 1983), which assesses the frequency of experiencing four stress-related feelings/thoughts in the last month.

*Hypertension* and *diabetes*. We also asked about personal health conditions including whether participants had ever been diagnosed with hypertension or Type 2 diabetes.

*Social support for PA* was measured using the scale developed by Sallis et al. (1987) that is correlated with PA (how often *family* or *friends* *encourage them to be active*, how often they *offered to do PA with them* and how often they *did PA with them*).

*Acculturation*. The four-item Brief Acculturation Scale for Hispanics (Norris et al., 1996) assessed participants’ language preferences in general (for reading and speaking), to speak at home, to think, and to speak with friends (1 = only English to 5 = only Spanish; a 3 was for both equally). All items were reverse coded and summed, with higher scores indicating higher acculturation.

*Socio-demographics*. Standard measures assessed age, sex/gender, race-ethnicity, education (which we dichotomized into “high school or less” and “more than high school”), current employment status (which we dichotomized into “working part- or full-time,” versus “not working”).

### Data Analysis

The analytic sample comprised participants from cohort 1 baseline and cohort 2 baseline who completed the health questionnaire, accelerometry, and biometrics (*n* = 726). First, we examined descriptives by cohort on sociodemographic factors, accelerometry, social support for PA, park measures, biometrics and physical health, and mental health.

For the first research question (factors associated with MVPA in this churchgoing Latino sample), we used linear regression models to test the associations between sociodemographic factors (age, sex, education, family size, work status, acculturation), social support for PA, BMI, and mental health on MVPA while controlling for cohort, accelerometer wear time, and season. For the second question (factors associated with self-reported park visits in this churchgoing Latino sample), we used linear regression models to test the associations between the same factors in the first research question plus park safety concerns on self-reported park visits, while controlling for cohort and season.

To answer the third question (are an individuals’ MVPA and park use associated with mental health and other health outcomes in this church-going Latino sample?), MVPA and self-reported park visits were including as primary independent variables, along with previously mentioned covariates [socio-demographics, social support for PA, and BMI (except in model predicting BMI)] and controls (cohort, accelerometer wear time, season). In the models predicting depression (PHQ-8), perceived stress, and BMI, linear regression models were used. Logistic regression models were used for dichotomous outcomes: having been diagnosed with hypertension and with type 2 diabetes.

We did not control for race/ethnicity as almost the entire sample is Latino (99%). In sensitivity analyses excluding BMI, estimates were similar, indicating that adjustment for BMI did not materially alter the observed associations. We retained BMI given its established importance as both a covariate and potential mediator in cardiometabolic outcomes and to facilitate comparison with prior literature. Analyses were conducted using SAS v9.4.

## Results

In the first cohort, 407 individuals from 6 churches completed the health survey, wore the accelerometer, and completed biometrics. In the second cohort, 319 individuals from 6 churches completed these measurements.

Overall, among the 726 persons in this analysis, the average age was 52.3 (SD13.8), 76% were women and 24% men, and 71% completed high school or less and 29% completed more than high school. A total of 56% reported working full or part-time, while 44% reported not working (Table 1). Most (89%) self-identified as of Mexican background, with 8% self-identifying as Central American and 3% other Latino subgroup. In terms of annual household income, 38% reported <$20,000; 34% reported between $20,000 and $39,999; and 29% reported >$40,000. The average minutes of weekly MVPA measured by accelerometer was 196.3 (SD 167.4) but there was a significant discrepancy between the cohort recruited in 2019, with only 183.1 (SD 147.3) weekly minutes compared to the 2023 cohort with 213.1 (SD = 188.9) weekly minutes. There was also a significant difference in average MVPA between males and females, [Male: 271.3 (SD = 173.3); Female: 173.2 (SD = 158.7)]. PA was highest in the spring and summer and lowest in the winter.

**Table 1:** Participant characteristics (*n* = 726)

Characteristics	Cohort 1, 2019 (*n* = 407)	Cohort 2, 2023 (*n* = 319)	Total (*n* = 726)
*Sociodemographic factors*			
Age, *mean (SD)*	52.1 (SD = 13.5)	52.5 (SD = 14.2)	52.3 (SD = 13.8)
Sex/Gender, *n (%)*			
Male	87 (21.4%)	84 (26.3%)	171 (23.6%)
Female	320 (78.6%)	235 (73.7%)	555 (76.5%)
*Race/Ethnicity,* * n (%)*			
Latino^a^	402 (99.3%)	318 (100.0%)	720 (99.6%)
Non-Latino White	2 (0.5%)	0	2 (0.3%)
Non-Latino American Indian or Alaska Native	1 (0.3%)	0	1 (0.1%)
Education,* n (%)*			
High school or less	287 (71.0%)	230 (73.3%)	517 (72.0%)
More than high school	117 (29.0%)	84 (26.8%)	201 (28.0%)
Family Size, *mean (SD)*	3.8 (SD = 2.0)	4.1 (SD = 2.2)	3.9 (SD = 2.1)
*Employment* *, n (%)*			
Not working	187 (46.0%)	133 (41.7%)	320 (44.1%)
Working part- or full-time	220 (54.1%)	186 (58.3%)	406 (55.9%)
Acculturation, *mean (SD)*	2.0 (SD = 1.2)	2.0 (SD = 1.1)	2.0 (SD = 1.1)
*Social support for PA*			
Family support for PA, *mean (SD)*	1.4 (SD = 1.1)	1.5 (SD = 1.1)	1.4 (SD = 1.1)
Friend support for PA, *mean (SD)*	1.0 (SD = 1.1)	1.0 (SD = 1.1)	1.0 (SD = 1.1)
*Accelerometry*			
Minutes in MVPA Per Week, *mean (SD)*	183.1 (SD = 147.3)	213.1 (SD = 188.9)	196.3 (SD = 167.4)
Male	243.4 (SD = 149.9)	300.3 (SD = 191.3)	Male: 271.3 (SD = 173.3)
Female	166.7 (SD = 142.4)	182.0 (SD = 178.3)	Female: 173.2 (SD = 158.7)
Wear hours per week, *mean (SD)*	96.6 (11.5)	93.7 (9.8)	95.3 (10.9)
*Season, n (%)*			
Fall	92 (22.6%)	90 (28.2%)	182 (25.1%)
Spring	173 (42.5%)	101 (31.7%)	274 (37.7%)
Summer	142 (34.9%)	116 (36.4%)	258 (35.5%)
Winter	0 (0.0%)	12 (3.8%)	12 (1.7%)
*Biometrics & physical health*			
BMI, *mean (SD)*	31.8 (SD = 7.1)	30.2 (SD = 5.2)	31.1 (SD = 6.3)
Hypertension, *n (%)*	98 (24.6%)	68 (21.5%)	166 (23.2%)
Diabetes, *n (%)*	74 (18.8%)	42 (13.6%)	116 (16.5%)
*Park measures*			
# Park visits per week, *mean (SD)*	1.3 (SD = 1.7)	1.4 (SD 1.8)	1.3 (SD = 1.8)
Perception of park safety, *mean (SD)* [higher = less safe]	1.1 (SD = 0.7)	1.0 (SD = 0.7)	1.0 (SD = 0.7)
*Mental health*			
PHQ-8 Score, *mean (SD)*	3.5 (SD = 4.5)	3.3 (SD = 4.1)	3.4 (SD = 4.3)
Perceived Stress Score, *mean (SD)*	4.4 (SD = 3.1)	5.0 (SD = 2.9)	4.7 (SD = 3.0)

^a^89% of participants self-identified as Mexican, Mexican American, or Chicano; 8% self-identified as Central American; and 3% self-identified as another Latino origin.

The average BMI was 31.1 (SD = 6.3), which is considered in the obese range. Hypertension diagnosis was reported by 23% and Type 2 diabetes by 17% of respondents. The average number of park visits per week was 1.8 and parks were considered relatively safe with a score of 1 (range 0–3) with the higher scores as more unsafe. Mental health and stress scores were favorable with an average PHQ-8 score of 3.4 (SD 4.3) out of 24 and PSS score of 1 (SD 1.1) from a range of 0–4. However, family and friend support for PA were relatively low, with average scores of 1.4 (SD = 1.1) and 1.0 (SD = 1.1), respectively.

Table 2 shows the factors associated with accelerometry measured MVPA. Age was negatively associated with MVPA (β= − 2.79, *p* < 0.0001) as was being female (β = − 87.10, *p* < 0.0001) and having a higher education level (β= − 43.10, *p* = 0.0139). Having higher PHQ8 scores (higher depressive symptoms) was associated with lower MVPA (β= − 6.02, *p* = 0.0004).

**Table 2:** Multivariable associations with accelerometer measured MVPA

Parameter	Estimate	Standard error	Pr > |t|
*Sociodemographics*			
Age	**− 2.79**	**0.52**	**< 0.0001**
Female	**− 87.11**	**15.29**	**< 0.0001**
> HS education	**− 43.10**	**17.46**	**0.0139**
Family size	1.23	3.12	0.6933
Working part- or full-time	− 0.56	13.70	0.9676
Acculturation score	1.92	6.88	0.7804
*Social support*			
Family support for PA	− 10.73	6.12	0.0800
Friend support for PA	− 3.88	6.06	0.5226
*Mental health*			
PHQ8 score (depression)	**− 6.02**	**1.70**	**0.004**
Perceived stress	3.36	2.33	0.1497
*Controls*			
Accelerometer wear hours per week	0.94	0.60	0.1192
BMI	0.32	1.00	0.7472
Cohort 1	**− 30.69**	**13.07**	**0.0192**
Cohort 2	0.0000000	.	.
Fall	76.32	50.14	0.1285
Spring	54.79	49.59	0.2697
Summer	89.55	49.71	0.0722
Winter	0.0000000	.	.
Intercept	284.07	86.12	0.0010

Bolded results indicate p < .05

Table 3 shows the factors associated with the number of park visits. Being employed was associated with 0.5 fewer park visits per week (*p* = 0.001). Family support for PA was associated with more park visits (β = 0.33, *p* < 0.0001), but friend support for PA was not significantly associated with park visits. More park visits were associated with lower levels of depression (β = − 0.05, *p* = 0.02). Having more concerns about park safety was also associated with fewer park visits (β = − 0.27, *p* = 0.02).

**Table 3:** Multivariable associations with self-reported park visits

Parameter	Estimate	Standard error	Pr > |t|
*Sociodemographics*			
Age	0.00	0.01	0.84
Female	− 0.30	0.18	0.11
> HS education	0.18	0.21	0.38
Family size	0.00	0.04	0.99
Working part- or full-time	**− 0.53**	**0.16**	**0.001**
Acculturation score	0.01	0.08	0.88
*Social support*			
Family support for PA	**0.33**	**0.07**	**< 0.0001**
Friend support PA	0.07	0.07	0.34
*Mental health*			
PHQ8 score	**− 0.05**	**0.02**	**0.02**
Perceived stress	0.037	0.03	0.19
**Park safety concerns**	**− 0.27**	**0.11**	**0.02**
*Controls*			
BMI	− 0.00	0.01	0.68
Cohort 1	− 0.12	0.16	0.44
Cohort 2	0.00	.	.
Fall	0.86	0.64	0.18
Spring	0.27	0.64	0.67
Summer	0.83	0.64	0.19
Winter	0.00	.	.
Intercept	1.12	0.89	0.21

Bolded results indicate p < .05

Table 4 confirms that park visits and MVPA were both independently associated with lower depression. Each minute of MVPA was associated with 0.003 lower points on the scale measuring depression (*p*<0.01). Each additional park visit was associated with 0.22 lower points on the PHQ-8 (*p *= 0.04). Park visits were also associated with 19% lower odds of Type 2 DM [OR = 0.81 (95% CI = 0.69, 0.95)]. Family support for PA was also associated with a lower risk of hypertension [OR = 0.77 (95% CI = 0.63, 0.96)].

**Table 4:** Multivariable associations with physical and mental health outcomes

	Depression (PHQ-8)			Perceived Stress			BMI			Hypertension		Type 2 diabetes	
	Beta	SE	*p*	Beta	SE	*P*	Beta	SE	*p*	OR	95% CI	OR	95% CI
*Primary independent variables*													
Acc MVPA minutes per week	**-0.003**	**0.001**	**< 0.01**	– 0.0004	0.001	0.63	– 0.0004	0.002	0.83	1.00	1.00, 1.00	1.00	1.00, 1.00
Park visits per week	**-0.22**	**0.10**	**0.04**	0.04	0.07	0.60	– 0.13	0.16	0.40	0.93	0.82, 1.06	**0.81**	**0.69**,** 0.95**
*Sociodemographics*													
Age	– 0.02	0.01	0.11	**– 0.03**	**0.01**	**< 0.01**	– 0.01	0.02	0.73	**1.09**	**1.06**,** 1.11**	**1.04**	**1.02**,** 1.06**
Female	0.34	0.43	0.43	0.30	0.31	0.34	1.14	0.65	0.08	0.76	0.44, 1.29	0.55	0.30, 1.02
Education: more than HS (vs. HS or less)	– 0.19	0.49	0.69	0.04	0.34	0.91	0.09	0.72	0.90	1.22	0.67, 2.21	0.70	0.35, 1.40
Family size	0.03	0.09	0.77	0.07	0.06	0.22	– 0.08	0.13	0.52	**1.15**	**1.04**,** 1.27**	1.03	0.92, 1.16
Working part- or full-time	– 0.94	0.38	**0.01**	– 0.47	0.27	0.08	– 0.08	0.56	0.89	0.88	0.56, 1.40	**0.42**	**0.25**,** 0.72**
Acculturation score	0.48	0.19	**0.01**	– 0.06	0.13	0.63	– 0.08	0.28	0.78	1.06	0.85, 1.32	0.96	0.75, 1.24
*Social Support*													
Family support for PA	– 0.29	0.17	0.10	– 0.03	0.12	0.83	0.01	0.26	0.97	**0.77**	**0.63**,** 0.96**	1.09	0.87, 1.38
Friend support for PA	– 0.04	0.17	0.79	0.16	0.12	0.17	– 0.34	0.25	0.17	1.08	0.88, 1.33	0.81	0.64, 1.04
*Controls*													
BMI	**0.08**	**0.03**	**< 0.01**	**0.05**	**0.02**	**0.02**	–			**1.04**	**1.01**,** 1.08**	**1.04**	**1.01**,** 1.08**
Cohort 1 (ref = Cohort 2)	– 0.06	0.36	0.87	**-0.55**	**0.26**	**0.03**	**1.33**	**0.54**	**0.01**	1.12	0.73, 1.74	1.53	0.93, 2.54
Acc Total Wear Hrs	0.001	0.02	0.98	0.003	0.01	0.77	– 0.004	0.03	0.86	0.99	0.97, 1.01	0.98	0.96, 1.00
Acc Wear Season: Fall	2.08	1.37	0.13	– 1.17	1.00	0.24	1.28	2.14	0.55	0.98	0.14, 6.76	1.05	0.11, 10.55
Acc Wear Season: Spring	1.05	1.36	0.44	– 1.08	0.99	0.28	1.85	2.12	0.38	1.25	0.18, 8.47	0.88	0.09, 8.66
Acc Wear Season: Summer	1.60	1.36	0.24	– 1.48	1.00	0.14	0.87	2.13	0.68	0.83	0.12, 5.65	1.16	0.12, 11.46
Acc Wear Season: Winter	REF			REF			REF			REF		REF	

Bolded results indicate p < .05

## Discussion

This study demonstrates important associations between park visits, objectively measured MVPA, and health outcomes among church-going Latino adults (*n* = 726) in high-poverty, urban areas of Los Angeles. Specifically, our findings indicated that more park visits per week and higher MVPA were both independently associated with lower depressive symptoms, while park visits were also independently associated with lower odds of Type 2 diabetes and family support for PA was associated with lower odds of hypertension. These results build on and extend prior work showing the potential benefits of park access and use for both physical and mental health, especially in underserved communities.

Importantly, self-reported number of weekly park visits had a stronger association than objectively measured MVPA with several outcomes, especially depression. This could be because park visits are more likely to reflect contact with nature, while MVPA could include all the PA associated with transport and even working as part of one’s job. There is increasing evidence that occupational PA does not confer the same health benefits, particularly cardiovascular, as leisure-time PA (Holtermann et al., 2012; Cillekens et al., 2022; Janssen & Voelcker-Rehage 2023). In a large study of Latino adults from four urban sites across the U.S., greater occupational PA was associated with shorter sleep duration, a risk factor for obesity and multiple adverse health outcomes (Tom et al., 2020). Latino adults with lower educational attainment often perform greater amounts of occupational PA that is strenuous but repetitive and not necessarily health-enhancing. Conversely, participants with higher education levels may have more sedentary employment, as suggested by our finding of a negative correlation between education and MVPA. Further, our finding of an independent association between park visits and mental health is supported by evidence that spending time in nature does have mental health benefits (Sundermann et al., 2023; Jimenez et al., 2021; Olszewska-Guizzo et al., 2022; Piva et al., 2024), and could be particularly important for individuals living in densely populated urban areas who face many daily stressors. Longitudinal studies during the COVID-19 pandemic found that spending time outdoors is associated with better mental health (Young et al., 2022) and more PA (Olivera Leon et al., 2024). In addition, urban parks have been found to be important places for social interactions for immigrant Latino populations (Vaughan et al., 2018; Derose et al., 2015), which could provide additional psychosocial benefits.

Depression and stress remain significant concerns within Latino communities, particularly among adults living in low-income urban areas. For example, financial strain among older Mexican American adults has been associated with high depressive symptoms (Hernandez et al., 2024), and depression has been found to be high among Latino adults with diabetes (Ezinne et al., 2025). Further, studies using the landmark Hispanic Community Health Study/Study of Latinos data found consistent associations between low income and high chronic stress and greater depression and anxiety symptoms and these were associated with cardiovascular health outcomes (Gallo et al., 2025). Although average PHQ-8 scores in our sample of churchgoing Latino adults were relatively low (~ 10% met criteria for major depression), the presence of even mild symptoms can affect daily functioning and chronic disease management. The observed associations of both MVPA and park visits with lower depressive symptoms emphasize the potential relevance of community and environmental supports for mental health in this population.

Other findings of note were that the average BMI in our sample was in the obese range, and the prevalence of hypertension and diabetes was elevated, underscoring the high cardiometabolic risk profile among churchgoing Latino adults in these communities and the need for community-based interventions. Although the average MVPA surpassed the adult recommended minimum of 150 min per week – with males exceeding this significantly – most participants, particularly those with obesity, still fell short of the 200–300 min per week recommended for weight management and broader health benefits (Donnelly et al., 2009). This could be due to the high proportion of women in the study, given that women had substantially lower MVPA than men, as seen with other racial/ethnic groups (Troiano et al., 2008), which may reflect greater household and caregiving responsibilities, differential social support, or occupational differences. The higher proportion of women in our sample than men is consistent with national trends showing that women in general (regardless of racial-ethnic group) are more likely to attend church than men (McClendon 2016) and previous faith-based studies (Flórez et al., 2020). Additional efforts may be needed to engage Latino men in faith-based studies. Nevertheless, church-based interventions that are family-oriented and sensitive to women’s roles and constraints may be particularly effective for increasing PA among Latino women, helping to address observed PA disparities while leveraging the trusted and accessible setting churches provide for women who often attend with their families.

Low levels of reported family and friend support for PA in this sample of churchgoing Latino adults highlight additional opportunities for social- and community-based interventions. Our results show that family support was associated with both more frequent park visits and lower hypertension, suggesting that interventions that simultaneously strengthen supportive environments and facilitate park engagement can have multiple benefits. Family support for PA may influence health through psychosocial and stress-related pathways that are partly independent of PA itself, such as promoting relaxation, social connection, and emotional support that buffer chronic stress and improve metabolic and cardiovascular regulation. The negative association between concerns about park safety and park visits suggests the need for ongoing local government investment in improving perceived and actual park safety and amenities.

Faith-based organizations – trusted and influential within Latino communities – may be well-positioned to promote not only PA, but also neighborhood park visits, providing support and organized programming that can benefit health. Findings from the Faith, Activity, and Nutrition (FAN) trial showed that an intervention developed through community-based participatory research and drawing on the social ecological model to target organizational and environmental changes at African American churches can successfully increase participants’ self-reported leisure-time PA (Wilcox et al., 2013) through increased social support and self-efficacy (Saunders et al., 2014). In Latino parishes, similar approaches could involve collaboration with priests or pastors, parish councils, and lay leaders to ensure that activities are culturally relevant, family-oriented, and responsive to congregants’ schedules and safety concerns. Partnering with local park departments to host family or ministry-sponsored park events may also foster greater trust and sustained participation and may be particularly effective in low-income Latino communities that face compounded health and resource inequities.

Building and sustaining trust with communities that face socioeconomic disadvantages and immigration-related stressors is increasingly challenging. Faith-based settings can serve as credible and culturally grounded partners for health promotion, offering infrastructure that reaches individuals and families who may not otherwise engage with formal health systems. This study represents an initial step in understanding how churches can collaborate with parks and public health agencies to create supportive environments for physical activity and social connection. Strengthening these multisectoral partnerships may be critical for improving health equity and fostering resilience in Latino urban communities.

Limitations of the study include its cross-sectional design, precluding causal inference; accelerometry data that only captured one week; and the potential non-representativeness of a sample primarily composed of (76%) churchgoing Latino women, which limits generalizability. There was also variation between the two cohorts due to the COVID-19 pandemic, which is known to have affected PA behavior and outdoor time (Neville et al., 2024; Cui et al., 2023; Nindenshuti & Caire-Juvera 2023; Mehraeen et al., 2023; Ng et al., 2022); In addition, hypertension and diabetes were assessed through self-report, and earlier studies using objective measures have found that Latino adults in the U.S. were unaware of their diabetes (Schneiderman et al., 2014, Barcellos et al., 2012) and hypertension (Egan et al., 2010), which, if true in our study, could bias our results. The measure of acculturation used (BASH) (Norris et al., 1996) accounts only for language preferences and not values, cultural beliefs and attitudes, identity, and time in the U.S., although in our sample, acculturation was highly correlated with nativity (*r* = 0.64, *p* < 0.0001). Also, we did not control for income because it was moderately correlated with education (*r* = 0.41, *p* < 0.0001), and we opted for education given a lower rate of missingness. Despite these limitations, our objectively measured and socially contextualized findings support further exploration of parks and PA – as well as the leveraging of faith-based settings – for improving both physical and mental health outcomes among Latino populations in urban settings.

## Conclusion

Park visits and PA are both health-promoting behaviors that are relevant across racial and ethnic groups. In this churchgoing Latino population living in low-income urban areas, we observed positive associations between mental health and MVPA and park visits. Although the cross-sectional nature of this study precludes conclusions about directionality or causality, these findings suggest that engagement in PA and park use may be important and independent correlates of mental health in this populations. Churches represent one potential setting through which PA and park-related activities could be encouraged, particularly given their social infrastructure and reach within Latino communities. Although prior research has demonstrated how MVPA can be increased among Latina churchgoing women through church-based exercise classes (Arredondo et al., 2017), additional research is needed to evaluate whether church-based efforts can effectively increase MVPA among men and park use among all genders and to determine their impact on health outcomes. Future studies using longitudinal or intervention designs could examine whether leveraging church infrastructure, in combination with community or public health partners, influences PA behaviors. Given relatively low reported support for PA from friends and family, additional community-level or media-based approaches may also warrant exploration. Overall, these findings highlight the need for cautious, theory-driven research to better understand how multisectoral interventions may support PA and park engagement and their potential implications for wellbeing.

## Footnotes

1 Several terms are used to describe people from Latin America or of Latin American descent – Latino/a or the “gender-neutral,” Latinx and Latine. “Hispanic” generally refers to people from Spanish-speaking countries. We use the term “Latino” because this is the broad term that our participants and community partners use to describe people from Latin America or of Latin American descent.
